# Supplementary material for: Post-transcriptional splicing can occur in a slow-moving zone around the gene
Source: eLife. 2024 Apr 5;12:RP91357. doi: 10.7554/eLife.91357 (PMC10997330; doi:10.7554/eLife.91357)
Supplement: Supplementary file 1. — (A) Dispersal and percent of splicing that occurs distally post-transcriptional, as assayed through RNA FISH. Each row indicates one probe set. (B) Percent of intermediate splicing products that splice in one order versus the other, indicating that splicing can generally occur in any order. [file elife-91357-supp1.docx]

## **Supplementary File 1**

Supplementary File 1A: Per probe set dispersal and percent of splicing that occurs distally post-transcriptional

| **Gene** | **Intron #** | **# of Probes** | **% coloc with exon** | **mean dispersal (um)** | **% of intensity that is not local to txn site** |
| --- | --- | --- | --- | --- | --- |
| CPS1 | 1/37 | 10 | 75.5814 | 0 | 0 |
| CPS1 | 13/37 | 12 | 88.2716 | 0.067485645 | 1.337543 |
| CPS1 | 21/37 | 24 | 74.71264 | 2.0966995 | 27.7981585 |
| CPS1 | 34/37 | 16 | 88.88889 | 0.03229656 | 1.952689 |
| EEF2 | 1/14 | 21 | 91.66667 | 0.93700431 | 15.69285 |
| EEF2 | 2/14 | 21 | 91.73554 | 0.41633783 | 6.482196 |
| EEF2 | 11/14 | 13 | 94.08867 | 1.67628235 | 24.386486 |
| TM4SF1 | 1/4 | 17 | 98.59155 | 0 | 0 |
| TM4SF1 | 3/4 | 24 | 94.78261 | 0.34731502 | 2.422979 |
| TM4SF1 | 4/4 | 24 | 87.84648 | 3.00480873 | 47.094267 |
| FKBP5** | 1/10 | 32 | 77.52809 | 1.2286272 | 22.451107 |
| FKBP5** | 3/10 | 32 | 94.64286 | 0.4514456 | 3.692255 |
| FKBP5** | 5/10 | 32 | 73.4375 | 2.852407 | 6.836791 |
| FKBP5** | 8/10 | 32 | 81.41593 | 1.4870159 | 33.077603 |
| FKBP5** | 9/10 | 14 | 93.89671 | 0.5677152 | 3.679758 |
| FKBP5** | 10/10 | 11 | 71.60494 | 0 | 0 |

** reflects values after 8 hours in dexamethasone

Supplementary File 1B: Percent of intermediate products that splice in one order versus the other

| **Gene** | **Intron A (5’)** | **Intron B (3’)** | **Total Captured Transcripts** | **Total Intermediate Products** | **% that splice Intron A first** | **% that splice Intron B first** |
| --- | --- | --- | --- | --- | --- | --- |
| EEF2 | Intron 1 | Intron 11 | 66272 | 427 | 42.85714286 | 57.14285714 |
| EEF2 | Intron 2 | Intron 1 | 55769 | 270 | 50.37037037 | 49.62962963 |
| EEF2 | Intron 2 | Intron 11 | 42077 | 190 | 63.68421053 | 36.31578947 |
| TM4SF1 | Intron 1 | Intron 4 | 20412 | 406 | 88.42364532 | 11.57635468 |
| TM4SF1 | Intron 1 | Intron 3 | 30345 | 129 | 58.91472868 | 41.08527132 |
| TM4SF1 | Intron 3 | Intron 4 | 26041 | 725 | 87.44827586 | 12.55172414 |
| TM4SF1 | Intron 4evens** | Intron 4odds** | 25726 | 122 | 60.6557377 | 39.3442623 |
| FKBP5 | Intron 3 | Intron 10 | 4534 | 213 | 19.24882629 | 80.75117371 |
| FKBP5 | Intron 3 | Intron 5 | 4260 | 164 | 65.24390244 | 34.75609756 |
| FKBP5 | Intron 1 | Intron 8 | 155 | 69 | 73.91304348 | 26.08695652 |
| FKBP5 | Intron 5 | Intron 8 | 2745 | 284 | 94.01408451 | 5.985915493 |
| FKBP5 | Intron 8 | Intron 9 | 3045 | 367 | 5.449591281 | 94.55040872 |
| FKBP5 | Intron 8$ | Intron 9$ | 2648 | 273 | 11.72161172 | 88.27838828 |
| FKBP5 | Intron 9 | Intron 10 | 2025 | 33 | 72.72727273 | 27.27272727 |
| FKBP5 | Intron 1.2 | Intron 1.1 | 4049 | 262 | 47.32824427 | 52.67175573 |
| CSP1 | Intron 1** | Intron 13** | 22960 | 31 | 48.38709677 | 51.61290323 |
| CSP1 | Intron 13 | Intron 21 | 17032 | 62 | 77.41935484 | 22.58064516 |
| CSP1 | Intron 13** | Intron 34** | 21977 | 47 | 53.19148936 | 46.80851064 |
| CSP1 | Intron 1 | Intron 21 | 20879 | 95 | 70.52631579 | 29.47368421 |
| CSP1 | Intron 21 | Intron 34 | 19775 | 76 | 25 | 75 |
| CSP1 | Intron 1 | Intron 34 | 13640 | 35 | 57.14285714 | 42.85714286 |

$ = dye swap experiment

** = measurements below noise threshold (see methods)
